# Blood oxygen level dependent magnetic resonance imaging for detecting pathological patterns in lupus nephritis patients: a preliminary study using a decision tree model

**DOI:** 10.1186/s12882-017-0787-z

**Published:** 2018-02-09

**Authors:** Huilan Shi, Junya Jia, Dong Li, Li Wei, Wenya Shang, Zhenfeng Zheng

**Affiliations:** 1 Department of Radiology, Tianjin Medical University General Hospital, No.154, Anshan Road, Heping District, Tianjin, People’s Republic of China; 2 Department of Nephrology, Tianjin Medical University General Hospital, No.154, Anshan Road, Heping District, Tianjin, People’s Republic of China

## Abstract

**Background:**

Precise renal histopathological diagnosis will guide therapy strategy in patients with lupus nephritis. Blood oxygen level dependent (BOLD) magnetic resonance imaging (MRI) has been applicable noninvasive technique in renal disease. This current study was performed to explore whether BOLD MRI could contribute to diagnose renal pathological pattern.

**Methods:**

Adult patients with lupus nephritis renal pathological diagnosis were recruited for this study. Renal biopsy tissues were assessed based on the lupus nephritis ISN/RPS 2003 classification. The Blood oxygen level dependent magnetic resonance imaging (BOLD-MRI) was used to obtain functional magnetic resonance parameter, R2* values. Several functions of R2* values were calculated and used to construct algorithmic models for renal pathological patterns. In addition, the algorithmic models were compared as to their diagnostic capability.

**Results:**

Both Histopathology and BOLD MRI were used to examine a total of twelve patients. Renal pathological patterns included five classes III (including 3 as class III + V) and seven classes IV (including 4 as class IV + V). Three algorithmic models, including decision tree, line discriminant, and logistic regression, were constructed to distinguish the renal pathological pattern of class III and class IV. The sensitivity of the decision tree model was better than that of the line discriminant model (71.87% vs 59.48%, *P* < 0.001) and inferior to that of the Logistic regression model (71.87% vs 78.71%, *P* < 0.001). The specificity of decision tree model was equivalent to that of the line discriminant model (63.87% vs 63.73%, *P* = 0.939) and higher than that of the logistic regression model (63.87% vs 38.0%, *P* < 0.001). The Area under the ROC curve (AUROCC) of the decision tree model was greater than that of the line discriminant model (0.765 vs 0.629, *P* < 0.001) and logistic regression model (0.765 vs 0.662, *P* < 0.001).

**Conclusions:**

BOLD MRI is a useful non-invasive imaging technique for the evaluation of lupus nephritis. Decision tree models constructed using functions of R2* values may facilitate the prediction of renal pathological patterns.

**Electronic supplementary material:**

The online version of this article (doi: 10.1186/s12882-017-0787-z) contains supplementary material, which is available to authorized users.

## Background

The kidney is one of the most frequently involved organs in systemic lupus erythematosus (SLE). Lupus nephritis (LN) is one of the most common secondary glomerulonephritis in China. Its clinical and pathological manifestations and prognosis are diverse and require specific therapeutic responses. A precise description of renal histopathological lesions and an appropriate classification of LN are essential for nephrologists to both guide treatment and predict the prognosis. The role of renal biopsy in diagnosis, treatment, management, and follow-up of LN is critical. Routine performance of renal biopsies in SLE patients with any signs of kidney disease has been advocated by some nephrologists [1, 2]. Although renal biopsies can provide the pathological information directly, being an invasive method, it entails a high risk of bleeding in SLE patients, particularly in those with coagulant function abnormality or renal atrophy.

Functional magnetic resonance imaging (fMRI) techniques such as BOLD MRI have shown considerable value in the evaluation of renal function in healthy patients and patients with renal diseases [3–6]. Since the first employment of BOLD MRI in the assessment of the renal oxygenation state in an animal model in 1996 [7], several studies have investigated the potential of BOLD MRI to identify various pathologic kidney conditions such as athermanous renovascular disease [8], ureteral obstruction [9], diabetes mellitus [10, 11], renal allograft [12], and chronic kidney disease [13, 14]. BOLD MRI is a noninvasive method that can assess the oxygen concentrations in regional tissues on the basis of the paramagnetic properties of deoxyhemoglobin as an endogenous contrast agent. However, few BOLD MRI and pathological studies on lupus nephritis have focused on the relationship between the pathological patterns and iconographic parameters since then.

We hypotheses that renal BOLD-MRI may show some kind of specific image patterns, which may corresponding to pathological types. The aim and motivation of our study were to explore relationship between renal R2* map characteristics and histological pattern. Due to the lack of relevant studies on renal BOLD MRI in patients with lupus nephritis, the main purpose of this study was to construct several algorithmic models by BOLD MRI index. By comparing the diagnostic capabilities of these models with each other, we will explore the potential of noninvasive fMRI techniques to diagnose pathological patterns in patients with lupus nephritis. .

## Methods

### Study protocol

#### Patients

This study was designed as an observational, open study. Patients were accrued from January 2015 to April 2015. Twelve patients underwent abdominal MRI using a 3.0 T scanner. This study was approved by *Tianjin Medical University General Hospital Ethical Committee*, and all participants gave informed consent before entering the study. Patients who fulfilled the 2012 International Collaborating Clinics classification criteria for systemic lupus erythematosus were included [15]. The disease activity was assessed with the SLE Disease Activity Index (SLEDAI) [16]. The serum creatinine value was used to calculate the eGFR with the Chronic Kidney Disease Epidemiology Collaboration (CKD-EPI) formula [17].

### Renal histopathology

Renal biopsy specimens were fixed in 4.5% buffered formaldehyde for light microscopy. Consecutive serial 2-um thick sections were used for histological staining. Stains used included hematoxylin and eosin, periodic acid-Schiff, silver methenamine, and Masson’s trichrome. Renal histopathological data of patients with renal biopsy-proven lupus nephritis were evaluated according to the ISN/RPS 2003 classification [18] by two experienced pathologists. The pathologists classified and scored the biopsies separately, blinded to patients’ data and scores of other observers. Patients with fewer than 10 glomeruli in their renal biopsies were excluded. In this study, cases of III + V were classified as class III and cases of IV + V were classified as class IV. Pathological parameter such as activity indices and chronicity indices were assessed by renal pathologists using a modified, previously reported system involving semi-quantitative scoring of specific biopsy features [19].

### Magnetic resonance imaging techniques

Magnetic resonance imaging was performed using a 3.0-T Imager (GE Discovery™ 750 3.0 T; General Electric; USA). BOLD-MRI were acquired using three consecutive parallel coronal slices for each side of kidney. The patients should be breath-hold of 20 s (in expiration) during MRI scanning. The scanner had a maximum gradient strength of 50mT/m and a slew rate of 200mT/m/s. A Torsopa eight-channel body coil was used. Images were acquired with a T_1_INPHASE + FAT sequence for morphologic evaluation using a T_1_ weighted fat-suppressed sequence. The field of view (FOV) was 380 × 380 mm, section thickness 7.0 mm, section width 1.0 mm, and the repetition time (TR)/ echo time (TE) 180/2.1. BOLD MRI was performed using a T2* spoiled gradient recalled echo (SPGR) sequence. Here, the field of view was 380 × 380 mm, the matrix 192 × 160, TR 100 ms, and TE 2.4 ms, 6.2 ms, 10.0 ms, 13.8 ms, 17.6 ms, 21.4 ms, 25.2 ms, and 29.0 ms. The echo number was 8.00, flip angle 35°, bandwidth 19.23 kHz, section thickness 7.0 mm, section width 1.0, section number 8, and scan time 25 s.

### Image analysis

R2* maps were constructed on an ADW 4.5 Workstation using the FUNCTOOL program. Three consecutive renal coronal anatomical planes were selected in each kidney. The entire kidney section, including both cortex and medulla, was selected; the renal collecting system and any incidental renal cysts were excluded. R2* values of each voxel of selected ROI were obtained by MATLAB 7.10 (MathWorks Inc.; Natick, MA, USA). From each larger region of interest (ROI), we randomly selected 100 groups of R2* values which included 100 consecutive voxels. We also calculated functions such as arithmetic mean, geometric mean, harmonic mean, range, standard deviation, quartile, skewness, kurtosis, variance, and sum in each group of R2* values. The calculated formulas were listed in Additional file 1. We constructed a line discriminate model, logistic regression model, and decision tree model; using groups of indexes. Subsequently, the predicted classifications were calculated by each group of indexes of R2* data. The final predicted classification of each patient was obtained according to the calculated probability of the entire 300 groups of the indexes of the R2* data.

The principle idea of our new methodology was to classify LN pathological type by using probability of algorithm model predictive classification. In other words, each sample of renal R2* data will generate one predictive classification independently and “votes” for the corresponding class. The majority of the votes decided the overall prediction. This aggregate vote of multiple samples was inherently less noisy and less susceptible to chance than a single sample output. This methodological idea was also similar to the random forest (RF), which mitigate the volatility due to small data and improves the robustness of predictions. [20]

### Statistical analyses

#### Algorithm models

Linear discriminant analysis (LDA) is a supervised categorical technique that maximizes group differences by creating a weighted linear combination of the discriminating variables. [Ref]. The original LDA has two derivation including fisher LDA (FLDA) and least square LDA (LSLDA). FLDA is based on Fisher-Rao’s criterion [21, 22], which is to find the projection *w* to maximize the objective function denoting the ratio of between-class to within-class variances.

$$ J(w)=\left|{w}^T{S}_bw\right|/\left|{w}^T{S}_ww\right| $$

The formula listed below indicates the discriminant functions.

$$ D={b}_1{X}_1+{b}_2{X}_2+\cdots +{b}_n{X}_n+c $$

Where *D* discriminant function, *b* the discriminant coefficient or weight for that variable, *X* discriminating variables, *c* a constant and *n* the number of predictor variables.

The binary logistic regression (LR) model is used because of the response variable takes just two values. This model is primarily used to identify the relationship between more independent variables (*X*_i_) and the dependent variable (*Y*) to predict the independent variables that are most influential on the dependent variable. The formulae listed below shows the relationship between response probability and the predictor variables.

$$ Logit\left({p}_i\right)=\log \left(\frac{p_i}{1-{p}_i}\right)={\beta}_{io}+{\beta}_{i1}{X}_{i1}+\cdots +{\beta}_{ik}{X}_{ik} $$

Where, $\left(\frac{p_i}{1-{p}_i}\right)$ is the ratio of the probability of one of the classification, *β*_0_, *β*_i_ are parameters to be estimated, and *p*_i_ is the response probability for *i*th group, *k* is number of variables. [23]

Decision tree is a simple algorithm technique to classify patterns in numerous categories. In this model, the relationship between data is represented in a tree structure, starting from a root node to different nodes via multiple branches and finally ending in some terminal nodes. In our current study, Chi-squared automatic interaction detection (CHAID) algorithm was used. By CHAID algorithm, the generated decision tree plots demonstrates relationship between split variables and associated related factors, which enables population subgroups with homogeneous to be revealed. Decision tree contains a group of multiple mutually exclusive pathway from root node to terminal nodes, which represents classification rules. [24]

#### Evaluation models

To verify the performance of our classification algorithm, a cross-validation procedure (leave-1-out method) was adopted. The classification accuracy was determined by computing (1) sensitivity (i.e. true positive predictions/total positive cases), (2) specificity (i.e. true negative predictions/total negative cases) (3) overall classification accuracy (i.e. total number of samples correctly classified/total number of samples) and (4) area under the receiver operating characteristic curve.

All analyses were carried out using the IBM® SPSS® Statistics software (version 22.0.0.0 IBM Corporation; Armonk, NY, USA) and MedCalc® statistics software (version 15.2; Mariakerke, Belgium). The statistical significance was determined at *P* < 0.05.

## Results

### General clinical and pathological data of patients with lupus nephritis

The clinical data of the 12 patients studied are summarized in Table 1.The average age of patients was 30.92±11.54 years (ranging from 15 to 52 years). The age of onset of all patents with lupus nephritis ranged from 0.25 to 180 months, and the average age was 49.19±60.76 month. The mean urine protein was 3.52±2.50 g/24 h (ranging from 0.34 to 7.14 g/24 h). There were 6 patients whose urine protein reached the levels common for nephrotic syndrome. The average score of SLEDAI was 20.58±6.49 and the activity in the majority of patients (9/12) was assessed as severe; 3 of the 12 patients showed moderate activity. According to CKD Kidney Disease Improving Global Outcomes (KIDIGO) stages guideline, 9 patients were classified as stage 1 CKD (CKD 1). There were only 2 patients in CKD 2 and 1 patient in CKD 3.

**Table 1:** Comparisons of clinical data and laboratory data in 12 patients with lupus nephritis

Clinical Indexes	Case 1	Case 2	Case 3	Case 4	Case 5	Case 6	Case 7	Case 8	Case 9	Case 10	Case 11	Case 12
Clinical Data												
Age (15–52 years)												
Time between diagnosis of LN (months)	36	180	48	84	156	24	24	2	2	33	0.25	1
Systolic blood pressure (mmHg)	130	130	130	160	140	110	120	140	100	150	120	120
Diastolic blood pressure (mmHg)	80	80	80	110	90	70	80	80	50	80	80	80
Nephrotic syndrome	+	–	–	+	+	+	–	–	–	+	–	+
SLEDAI	12	23	18	33	25	19	14	26	17	12	27	21
Laboratory data												
Hemoglobin (g/l)	78	103	116	100	131	138	105	81	98	133	92	108
Urine protein (g/24 h)	6.79	0.34	1.46	4.76	7.14	6.51	1.15	2.08	0.81	4.67	1.87	4.63
Serum creatinine (umol/L)	62	43	59	93	32	27	56	73	47	37	136	56
eGFR (ml/min/1.73 m2)	98	113	105	62	108	124	113	80	120	113	39	131
Serum albumin (g/dl)	16	34	33	30	16	26	36	21	29	26	28	12
anti-ds-DNA	+	–	+	+	+	+	+	+	+	–	+	+
anti-Sm	–	+	–	–	–	–	–	+	+	–	+	–
anti-Ro52	–	+	+	–	–	+	+	–	–	+	+	+
anti-SSA	+	–	+	–	+	+	–	–	–	+	+	+
anti-SSB	–	–	–	–	–	–	+	–	+	+	+	–
anti-RNP	+	+	–	–	+	–	–	+	–	+	+	+
anti-cardiolipin antibody	–	–	–	–	–	–	–	–	–	–	+	–
C3 (g/l)	59.2	72.3	51.6	49.2	55.83	53.7	67.4	35.8	33.1	57.7	26.3	28.5
C4 (g/l)	16.2	15.7	11.6	11.1	7.82	5.06	13.4	1.76	2.67	14.2	3.3	3.76
ESR (mm/h)	46	29	35	35	39	45	37	53	52	44	40	48

*ANA* anti-nuclear antibodies, *RNP* ribonucleoprotein, *SLEDAI* systemic lupus erythematosus disease activity index, *SSA* Sjogren’s syndrome A antigen, *SSB* Sjogren’s syndrome B antigen, *eGFR* estimated glomerular filtrate

The renal histopathological features of patients are listed in Table 2. According to the 2003 ISN/RPS classification system for lupus nephritis, the lupus nephritis III and IV were further divided in three subgroups according to activity and chronicity of lesions; that is, active lesion only (A), both active and chronic lesions (A/C), and chronic lesions only (C). Five patients were classified as class III (including 3 as class III + V) and 7 patients as class IV (including 4 as class IV + V). The average activity and chronicity indexes were 8.00±2.37 and 2.33±0.49, respectively.

**Table 2:** Comparisons of renal pathological parameter scores in 12 patients with lupus nephritis

Light microscopy	Case 1	Case 2	Case 3	Case 4	Case 5	Case 6	Case 7	Case 8	Case 9	Case 10	Case 11	Case 12
Number of glomeruli	14	28	41	16	21	16	17	27	21	15	15	21
Activity Index												
Glomerular cell proliferation	2	2	2	2	1	1	1	1	1	2	2	2
Leucocyte exudation	1	2	1	2	2	1	2	2	0	1	1	1
Karyorrhexis and fibrinoid necrosis	1	2	1	0	1	0	0	1	0	0	1	2
Cellular crescents	2	1	3	2	1	0	1	1	1	3	1	2
Hyaline deposits	1	2	2	2	2	1	1	1	1	1	2	2
Interstitial inflammation	2	2	1	1	1	1	1	1	1	1	2	2
Al score	9	11	10	9	8	4	6	7	4	8	9	11
Chronicity Index												
Glomerular sclerosis	1	1	0	0	0	0	0	1	0	0	0	0
Fibrous crescents	0	0	0	0	0	0	0	0	0	0	0	0
Tubular atrophy	1	1	1	1	1	1	1	1	1	1	2	1
Interstitial fibrosis	1	1	1	1	1	1	1	1	1	1	1	1
Cl score	3	3	2	2	2	2	2	3	2	2	3	2
Pathological diagnosis	IV-G (A/C) + V	IV-G (A/C) + V	IV-G (A/C)	IV-G (A/C) + V	III-(A/C) + V	III-(A/C) + V	III-(A/C) + V	III-(A/C)	III-(A/C)	IV-G (A/C)	IV-G (A/C)	IV-G (A/C) + V

All patients received oral prednisone therapy. Four patients received pulse intravenous cyclosphosphamide (600–800 mg per month). Three patients received mycophenolate mofetil, 1 patient received tacrolimus, and 4 patients received leflunomide.

### BOLD MRI and pathological images of patients with lupus nephritis

**Fig. 1:**
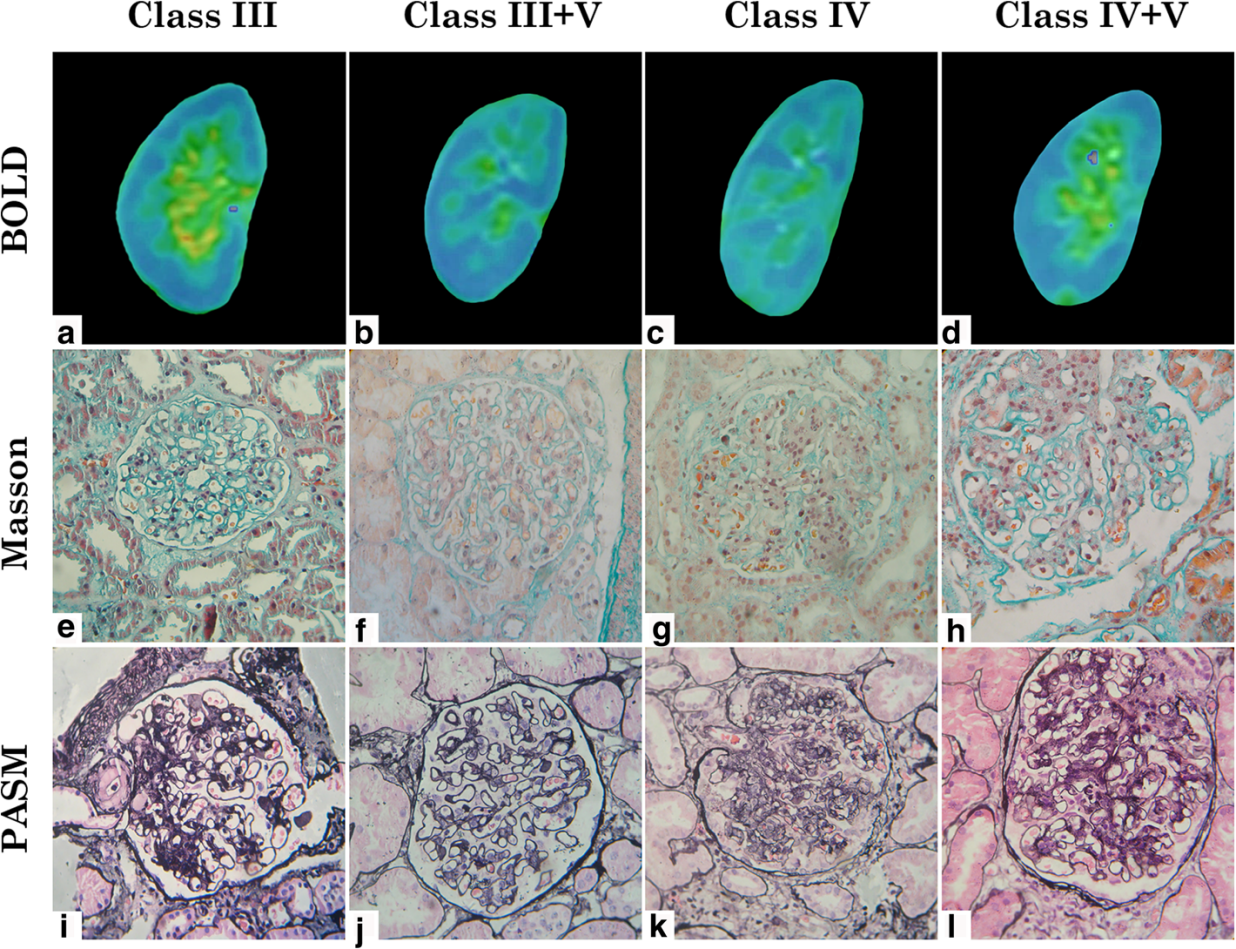
BOLD-MRI and renal pathological pictures of kidneys. Representative magnetic resonance images of a III type LN (**a**,**e**,**i**), III + V type (**b**,**f**,**j**), IV type (**c**,**g**,**k**) and IV + V type (**d**,**h**,**l**) LN patients. The BOLD-MRI pictures were expressed as pseudo-color maps. For example, blue represents the areas of lowest R2* values and oxyhemoglobin levels, whereas green, yellow, and red, in that order, represent increasing R2* values and higher oxyhemoglobin levels on the colored R2* map

Four groups of BOLD MRI and pathological images were obtained from 4 patients according to pathological diagnosis of lupus nephritis. Coronal planes of patients’ right kidneys were selected for BOLD MRI analysis. Renal biopsy stained sections were obtained for histopathological analysis. Using Masson’s thrichrome and silver methenamine stained sections, we differentiated four types of renal pathological diagnosis including class III, class III + V, class IV and class IV + V. See Fig. 1.

### Discrimination models or formulas for patients with lupus nephritis

We constructed two decision tree models which were used to distinguish the main and sub-classes of lupus nephritis (See Additional files 2 and 3).

The decision tree model distinguishing type III and IV LN in Additional file 2 shows the 3 level CHAID tree with a total of 42 nodes, of which 31 were terminal nodes. Six major predictor variables reached significance to be included in this model; including harmonic mean, quartile, standard deviation, range, kurtosis, and geometric mean. The first level of the tree was split into 7 initial branches, according to the user-specified first level of harmonic mean. Quartile, standard deviation, kurtosis, and range were shown to be the next predictor variables for each of the harmonic mean splits in the first level. Subsequently, range, harmonic mean, and geometric mean were the most prominent variables in the third level of the tree.

Two Fisher’s linear discriminants formulas and one Logistic regression formula were listed below for classification of the main class of pathological patterns.

Fisher’s linear discriminant formula:

$$ {\displaystyle \begin{array}{l} TypeIII=- 24.065+ 1.858\times harmonic\ mean+ 1.015\times kurtosis\\ {}+ 0.516\times range- 0.310\times skewness+ 2.964\times \\ {} standard\ deviation\end{array}} $$

$$ {\displaystyle \begin{array}{l} TypeIV=- 21.055+ 1.695\times harmonic\ mean+ 1.119\times kurtosis\\ {}+ 0.293\times range- 0.680\times skewness+ 3.715\times \\ {} standard\ deviation\end{array}} $$

Logistic regression formula:

$$ {\displaystyle \begin{array}{l} Logit(P)= 3.354- 0.166\times harmonic\ mean+ 0.117\times kurtosis\\ {}- 0.238\times range- 0.377\times skewness+ 0.820\times \\ {} standard\ deviation\end{array}} $$

Binary logistic regression analysis showed that a combination of five variables constructed formula to classify the pathological pattern with type III and type IV in LN patients.

### Evaluation of models or formulas of patients with lupus nephritis

**Fig. 2:**
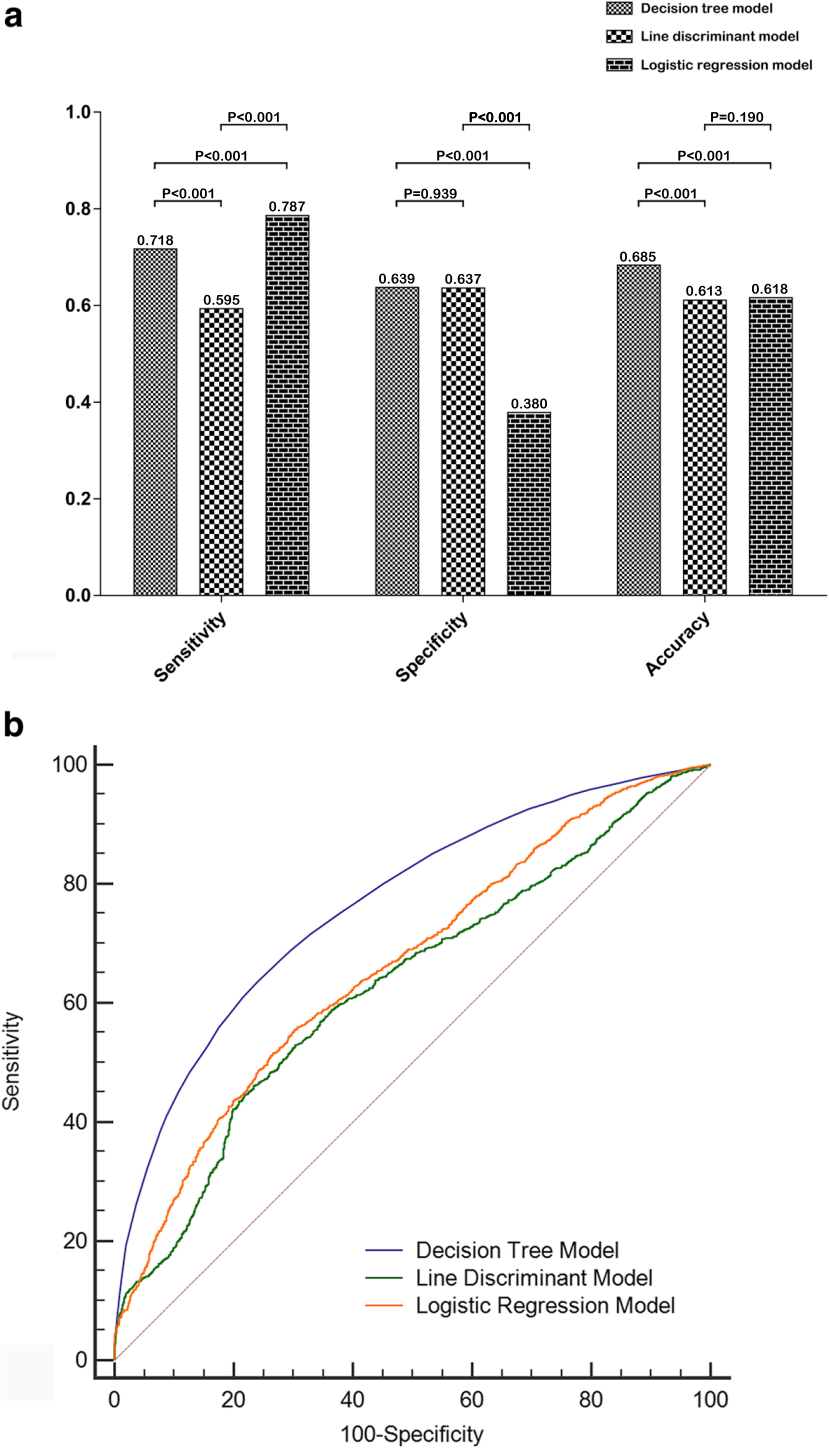
Decision tree model for predicting class III and class IV of lupus nephritis with CHIAD algorithm

For the evaluation of the three predictive models, by using 3600 groups R2* data in entire 12 patients, several indexes of diagnosis test were employed such as sensitivity, specificity, and accuracy. The logistic regression model had a good sensitivity (78.71%), but very poor specificity (38.0%). Conversely, the line discriminant model had a poor sensitivity (59.48%), but a better specificity (63.73%). The decision tree model had a better sensitivity than the line discriminant model (71.87% vs 59.48%, *P* < 0.001) and a higher specificity than the logistic regression model 63.87% vs 38.0%, *P* < 0.001). In addition, the accuracy and Youden’s indexes were highest in the decision tree model (68.5% and 0.3568), compared with line discriminant model (61.25% and 0.2321) and Logistic regression model (61.75% and 0.1671). Moreover, the diagnostic utility of the three models were evaluated by comparing it with the area under the ROC curve (AUROCC). The AUROCC of the decision tree model was greater than those of the line discriminant model (0.765 vs 0.629, *P* < 0.001) and logistic regression model (0.765 vs 0.662, *P* < 0.001). The AUROCC of the logistic repression model was also greater than that of line discriminant model (0.662 vs 0.629, *P* < 0.001) (see Fig. 2 and Table 3).

**Table 3:** Comparisons of predictive capability of the three algorithm models

Diagnosis Test Parameters	Decision Tree Model	Line Discriminant Model	Logistic Regression Model
Sensitivity	0.718^a,b^	0.595^c^	0.787
Specificity	0.639^b^	0.637^c^	0.380
Accuracy	0.685^a,b^	0.613	0.618
AUROCC	0.765^a b^	0.629^c^	0.662

*AUROCC* area under the ROC curve

^a^ Decision tree model vs Line discriminant model, *p* < 0.001

^b^ Decision tree model vs Logistic regression model, *p* < 0.001

^c^ Line discriminant model vs Logistic regression model, *p* < 0.001

The predicted primary classification of all 12 patients by three models was listed in Table 4. According to the calculated probabilities of all groups of R2* data, all predictive pathological classifications were corrected in the decision tree model. Compared with the decision tree model, only 8 predictive pathological classifications were corrected in line discriminate model. The four false predictive classifications were cases 2, 3, 4, and 9. There were also 4 false predictive pathological classifications, including cases 2, 5, 8, and 9 in the logistic regression model. We also constructed two decision tree models for discriminating sub-class of patients (see Additional file 3). Eleven predictive pathological classifications were corrected after comparing them with pathological findings in renal biopsies. Only in case 3, the predictive pathological classification (IV-G (A/C) + V) was false (see Table 5).

**Table 4:** Comparisons of primary renal pathological patterns predicted by the three algorithm models on the basis of predicted probability of R2* data

Case Number	Pathological Diagnosis	Predicted by Decision Tree Model (percentage/number, %/n)		Decision Tree Mode Result	Predicted by Line Discriminate Model (percentage/number, %/n)		Line Discriminate Mode Result	Predicted by Logistic Regression Model (percentage/number, %/n)		Logistic Regression Mode Result
		III Type	IV Type		III Type	IV Type		III Type	IV Type	
Case 1	IV	25% (74)	75% (226)	IV	15% (44)	85% (256)	IV	3% (8)	97% (292)	IV
Case 2	IV	42% (125)	58% (175)	IV	88% (263)	12% (37)	III	56% (176)	44% (124)	III
Case 3	IV	40% (119)	60% (181)	IV	62% (186)	38% (114)	III	32% (95)	78% (205)	IV
Case 4	IV	34% (103)	66% (197)	IV	63% (188)	37% (112)	III	38% (113)	62% (187)	IV
Case 5	III	55% (166)	45% (134)	III	65% (195)	35% (105)	III	37% (112)	63% (188)	IV
Case 6	III	60% (180)	40% (120)	III	81% (243)	19% (57)	III	55% (164)	45% (136)	III
Case 7	III	74% (221)	26% (79)	III	88% (263)	12% (37)	III	70% (209)	30% (91)	III
Case 8	III	73% (218)	27% (82)	III	65% (194)	35% (106)	III	24% (71)	76% (229)	IV
Case 9	III	58% (173)	42% (127)	III	20% (61)	80% (239)	IV	5% (14)	95% (286)	IV
Case 10	IV	23% (70)	76% (230)	IV	44% (133)	56% (167)	IV	17% (50)	83% (250)	IV
Case 11	IV	22% (67)	78% (233)	IV	1% (4)	99% (296)	IV	0% (1)	100% (299)	IV
Case 12	IV	11% (34)	89% (266)	IV	11% (33)	89% (267)	IV	1% (4)	99% (296)	IV

**Table 5:** Comparisons of secondary renal pathological patterns predicted by the decision tree models on the basis of predicted probability of R2* data

Case Number	Pathological Diagnosis	Predicted Primary Class (percentage/number, %/n)		Decision Tree Model Result	Predicted Secondary Class (percentage/number, %/n)		Decision Tree Model Result
		III Type	IV Type		Homogeneity	Heterogeneity	
Case 1	IV-G (A/C) + V	25% (74)	75% (226)	IV	17% (50)	83% (250)	IV-G (A/C) + V
Case 2	IV-G (A/C) + V	42% (125)	58% (175)	IV	5% (16)	95% (284)	IV-G (A/C) + V
Case 3	IV-G (A/C)	40% (119)	60% (181)	IV	35% (104)	65% (196)	IV-G (A/C) + V
Case 4	IV-G (A/C) + V	34% (103)	66% (197)	IV	30% (89)	70% (211)	IV-G (A/C) + V
Case 5	III-(A/C) + V	55% (166)	45% (137)	III	20% (61)	80% (239)	III-(A/C) + V
Case 6	III-(A/C) + V	60% (180)	40% (120)	III	6% (19)	94% (281)	III-(A/C) + V
Case 7	III-(A/C) + V	74% (221)	26% (79)	III	13% (39)	87% (261)	III-(A/C) + V
Case 8	III-(A/C)	73% (218)	27% (82)	III	60% (179)	40% (121)	III-(A/C)
Case 9	III-(A/C)	58% (173)	42% (127)	III	93% (280)	7% (20)	III-(A/C)
Case 10	IV-G (A/C)	23% (70)	76% (230)	IV	64% (192)	36% (108)	IV-G (A/C)
Case 11	IV-G (A/C)	22% (67)	78% (238)	IV	93% (279)	7% (21)	IV-G (A/C)
Case 12	IV-G (A/C) + V	11% (34)	89% (266)	IV	19% (58)	81% (242)	IV-G (A/C) + V

## Discussion

BOLD MRI technique was initially used to analyze oxygen metabolism for central nerves system. In recent decades, it was clinically available for renal imaging research because of several advances in fMRI technique. In the last decade, more and more studies focused on renal BOLD MRI to detect a variety of renal disorders or pathophysiological conditions such as atherosclerotic renal artery stenosis [25], chronic kidney disease [13, 14], renal transplantation [26], and diabetic nephropathy [10, 11]. Although these recent studies provided much useful information regarding renal BOLD MRI, there was not enough information about BOLD MRI used to classify and monitor renal manifestation of lupus nephritis. More recently, Li et al. reported renal oxygenation characteristics by using BOLD MRI technique in a group of lupus nephritis patients [27]. In this study, they explored the discrimination of renal R2* values in different pathological type of lupus nephritis. They found a lower R2* value in mixed proliferative and membranous LN (class III + V and IV + V) than in pure proliferative LN (class III and IV) or pure membranous LN (class V). However, they did not investigate how to differentiate between different pathological patterns of LN by using this noninvasive technique. To our best knowledge, there are no relevant studies on the potential of fMRI to predict pathological patterns of LN. Our study was a preliminary research in exploration with available models.

Before we developed these available mathematical models, a new method was adopted to acquire R2* values in kidneys of LN patients. Three consecutive coronal anatomical planes were selected as sampling planes in each kidney. A larger region of interest (ROI) which included renal cortex and medulla (excluding the renal collecting system and any incidental renal cysts) was selected. All pixels in the selected ROIs were converted to R2* values in each coronal planes. One hundred groups of data which contained 100 R2* values in each group were randomly selected in each ROI. Finally, a series of indexes of characteristics R2* data was calculated for preparing arithmetic models. There were three reasons why we adopted this novel method. Firstly, due to difference in oxygen supply and metabolic rate between renal cortex and medulla, levels of R2* vary gradually from the cortex to the medulla, reaching a most hypoxic zone in the deepest sections of the medullary pyramids. Non-equilibrium renal oxygen distribution along the nephron produces considerable spatial heterogeneity [28]. In addition, the size of ROIs acquired from renal images can impact accuracy, precision and reproducibility of R2* values. Larger ROIs are prone to lower variation of R2* values and less noisy mean values, but may underestimate R2* values in medulla [29]. On the other hand, smaller ROIs tend to be skewed by spatial and temporal heterogeneity of oxygen distribution in kidney [30]. Ebrahimi et al. reported that R2* obtained using the manual ROI method with small ROIs showed larger R2* variation than manual ROI method with larger ROIs, suggesting lower reproducibility of the former method [31]. Considering our research reproducibility, we selected a manual larger ROI method. Secondly, the conventional renal BOLD measurement method quantified R2* values in renal cortex and medulla. However, it is difficult to distinguish renal cortex and medulla on MR imaging, especially in serious renal disorders. In order to solve this problem, we did not strictly distinguish renal cortex and medulla where were the locations of ROIs. Conversely, plenty of randomized and equal sized R2* data were selected in all larger ROIs. We thought that numerous sampling might represent the characteristics of renal oxygenation distribution with less sampling errors. Thirdly, the majority of previous renal BOLD studies focused on measurement of average R2* values. No relevant studies explored the values of other data indexes such as harmonic means, standard deviation, skewness, etc. These indexes may provide more information about renal R2* values, contributing to the analysis of renal oxygenation.

We developed three arithmetic models to predict pathological patterns in LN patients. The evaluation analysis shows that decision tree model has the best diagnostic sensitivity and specificity as well as the highest accuracy and Youden’s index. We also validated the three models by using whole sampling data. Decision tree model showed better prediction capability of distinguishing pathological patterns than the other two models. Compared with traditional statistical predictive models, the decision tree model showed advantages over traditional predictive modeling procedures based on their ease of interpretability by nonstatisticians. One of the outstanding advantages of decision tree analysis is that it can visualize the relationship pathways with a tree image. [32]. In our current study, final predictive pathological diagnosis could be easily gained by means of assignment rules of decision tree after the sampling data of R2* values were given. However, the final predictive results should be calculated by means of formulas of line discriminant or logistic regression models after the same data were given. Another advantage of decision tree analysis concerns sampling data characteristics. In conventional statistic predictive models such as line discriminant analysis or logistic regression analysis, sampling data should be linear, exclusive, and normal distributive. However, sampling data which were acquired from the renal cortex or medulla region were not all distributive. For example, R2* date from medullary region were characterized by Gamma distribution function. It was not suitable to analyze these data by line discriminant analysis and logistic regression analysis [31].

Renal prognosis and outcomes may depend on disease activity and renal pathological lesion in patients with LN. Clinically, several scales of lupus nephritis such as SLEDAI 2000 [33] or BILAG 2004 [34] was widely used to assess disorder activity. However, the evaluation of renal lesion still depends on renal biopsy. Because different pathological patterns of LN may lead to different renal prognoses and therapy strategies, it is still critical that bioptic examinations of LN patients are performed. Yu et al. reported that interstitial lesions were significantly more severe in class IV LN, compared with moderate in class III and mild in class II and V. Interstitial infiltration, tubular atrophy, and interstitial fibrosis were significant independent risk factors for renal outcome [35]. Bao et al. compared the classical therapy strategy and multi-target therapy strategy on class IV + V LN. Ten of 20 patients in the multi-target therapy group and 1 of 20 patients in the intravenous cyclophosphamide group achieved complete remission at 6 months. The number of completed remission patients added to 13 and 3 in the multi-target therapy group and in intravenous cyclophosphamide group at 9 months, respectively [36]. Since renal biopsy is invasive, LN patients tend to have a great risk of bleeding due to coagulant function abnormality or Intolerability for renal biopsies of some patients with atrophic kidneys. If the pathological diagnosis could not be provided by renal biopsy, it was difficult to evaluate the prognosis and to devise a reasonable treatment strategy for the concerned LN patient. Our current study may provide a practical method for these patients. Another important issue concerning LN was histological transformations. Greloni et al. studied 45 LN patients who underwent at least two renal biopsies. Of the 71 repeat biopsies, pathological transformation occurred in 39 cases (54.9%) [37]. Although renal biopsy can provide the pathological information directly, it is not ideal for follow-ups. Therefore, it seems critical to find a noninvasive and effective method to dynamically evaluate renal pathological changes in patients with LN. Our decision tree models of BOLD MRI may provide a possible noninvasive method to assess renal histological transformations.

Our primary exploration study did have obvious limitations such as small sample size and narrow spectrum of clinical and histologic characteristics. Firstly, the study lacked the class II and VI LN patients because pathological injuries were too mild or extensive to undergo renal biopsy. The lack of class V LN patients data lead to our decision tree model without predictive capability in non-proliferative LN patients. The including patients were not representative of total population because of well-preserved GFR. It is well known that the eGFR will be lower when the renal pathological injury was extensive and active. Sometimes, some LN patients needed to undergo hemopurification therapy. Secondly, the R2* map may not evaluate the extent of histopathological injury. We found that lower R2* values were detected in extensive proliferative LN patients’ renal tissues. However, lower R2* values were also detected in control group (healthy volunteers group). [38]. Thirdly, we did not investigate other MRI index such as ADC, which was deemed to useful factor for prediction renal tissue fibrosis [39]. The lack of a standardized pre-study protocol was definitely another major limitation. We have already considered the multiple factors which can impact on renal R2* values. On one hand, not only we should consider many influenced factors as possible as we can, but also we should clarify the importance degree of each factor affecting on renal R2* values in order to map out reasonable standardized protocol. To our best knowledge, these possible influenced factors have not studied clearly in the published literature. On the other hand, lupus nephritis is not a common glomerulonephritis like IgA nephropathy. Standardizing lupus nephritis pathophysiological condition may decrease the qualified study samples. One of the feasible way was to analyze these factors by using well-designed research approach and data mining technique such as canonical correlation analysis and structural equation model (SEM).

## Conclusions

BOLD MRI is a useful non-invasive imaging technique for evaluating renal diseases. Decision tree models constructed by data characteristics of R2* values may facilitate predicting renal pathological patterns. More patients with diverse renal pathological patterns and more indexes of functional MRI are required to construct a widely used and robust predictive model.

## Additional files

Additional file 1: Calculate formula for R2* values statistic parameter, Detailed algorithm formula for statistic parameter including arithmetic mean, geometric mean, harmonic mean, range, standard deviation, quartile, variance, sum, skewness and kurtosis. (DOCX 36 kb)

Additional file 2: Decision tree model for predicting class III and class IV of lupus nephritis with CHIAD algorithm. (PDF 11.4 mb)

Additional file 3: Decision tree model for differentiating lupus nephritis sub-class patterns of renal pathology with CHIAD algorithm. **a** Class III vs class III + V. **b** class IV vs class IV + V. (PDF 21.5 mb)

## Abbreviations

BOLD: Blood oxygen level dependent
CHAID: Chi-squared automatic interaction detection
CKD-EPI: Chronic kidney disease epidemiology collaboration
fMRI: Functional magnetic resonance imaging
FOV: Field of view
KIDIGO: Kidney disease improving global outcomes
LN: Lupus nephritis
MRI: Magnetic resonance imaging
ROI: Region of interest
SLE: Systemic lupus erythematosus
SLEDAI: SLE Disease activity index
SPGR: Spoiled gradient recalled echo
TE: Echo time
TR: Repetition time
