# Supplementary material for: Blood oxygen level dependent magnetic resonance imaging for detecting pathological patterns in lupus nephritis patients: a preliminary study using a decision tree model
Source: BMC Nephrol. 2018 Feb 9;19:33. doi: 10.1186/s12882-017-0787-z (PMC5806290; doi:10.1186/s12882-017-0787-z)
Supplement: Supplementary file 1 — Calculate formula for R2* values statistic parameter, Detailed algorithm formula for statistic parameter including arithmetic mean, geometric mean, harmonic mean, range, standard deviation, quartile, variance, sum, skewness and kurtosis. (DOCX 36 kb) [file 12882_2017_787_MOESM1_ESM.docx]

Accessory document: calculate formula for statistic parameter

Arithmetic mean: $\overline{x}=\frac{\sum_{i=1}^{n} x_{i}}{n}$

Geometric mean:

Harmonic mean: $H=\frac{n}{\sum_{i=1}^{n} \frac{1}{x_{i}}}$

Range: $\omega=X_{H}-X_{L}$

Standard deviation: $\sigma=\sqrt{\frac{1}{n}\sum_{i=1}^{n} (x_{i}-\overline{x})^{2}}$

Quartile: $L_{p}=n\left(\frac{p}{100}\right)$

Variance: $\sigma^{2}=\frac{\sum_{i=1}^{n} (x_{i}-\overline{x})^{2}}{n}$

Sum: $\mu=\sum_{i=1}^{n} x_{i}$

Skewness: $g_{1}=\frac{\frac{1}{n}\sum_{i=1}^{n} (x_{i}-\overline{x})^{3}}{\left(\frac{1}{n}\sum_{i=1}^{n} (x_{i}-\overline{x})^{2}\right)^{3/2}}$

Kurtosis: $g_{2}=\frac{\frac{1}{n}\sum_{i=1}^{n} (x_{i}-\overline{x})^{4}}{\left(\frac{1}{n}\sum_{i=1}^{n} (x_{i}-\overline{x})^{2}\right)^{2}}$
