# Supplementary figures and images for: Blood oxygen level dependent magnetic resonance imaging for detecting pathological patterns in lupus nephritis patients: a preliminary study using a decision tree model
Source: BMC Nephrol. 2018 Feb 9;19:33. doi: 10.1186/s12882-017-0787-z (PMC5806290; doi:10.1186/s12882-017-0787-z)

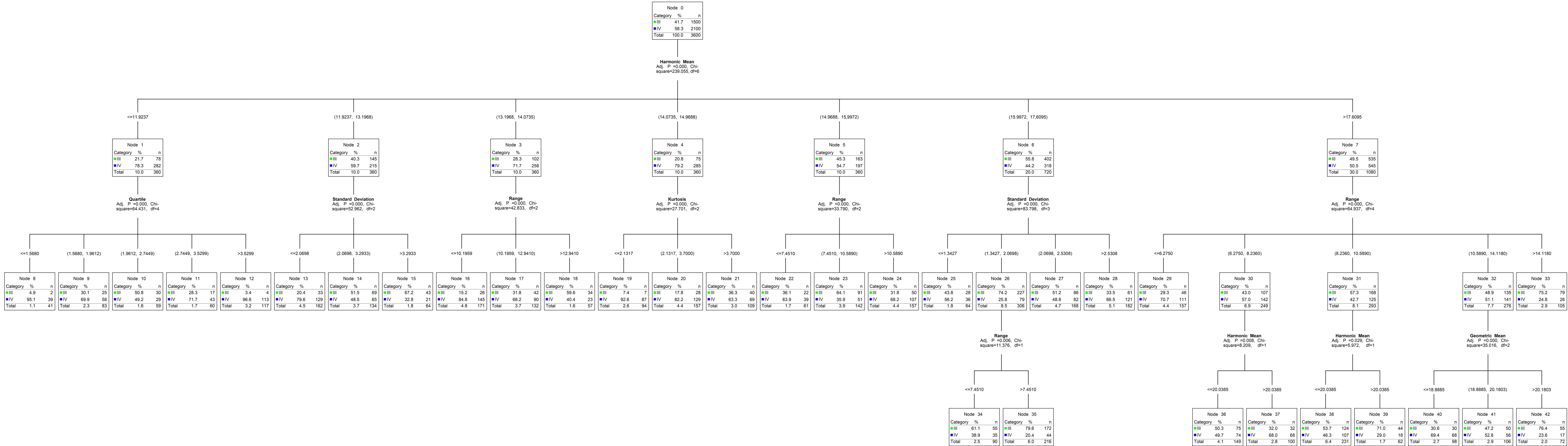

Supplement: Supplementary file 2 — Decision tree model for predicting class III and class IV of lupus nephritis with CHIAD algorithm. (PDF 11.4 mb) [file 12882_2017_787_MOESM2_ESM.pdf]

a

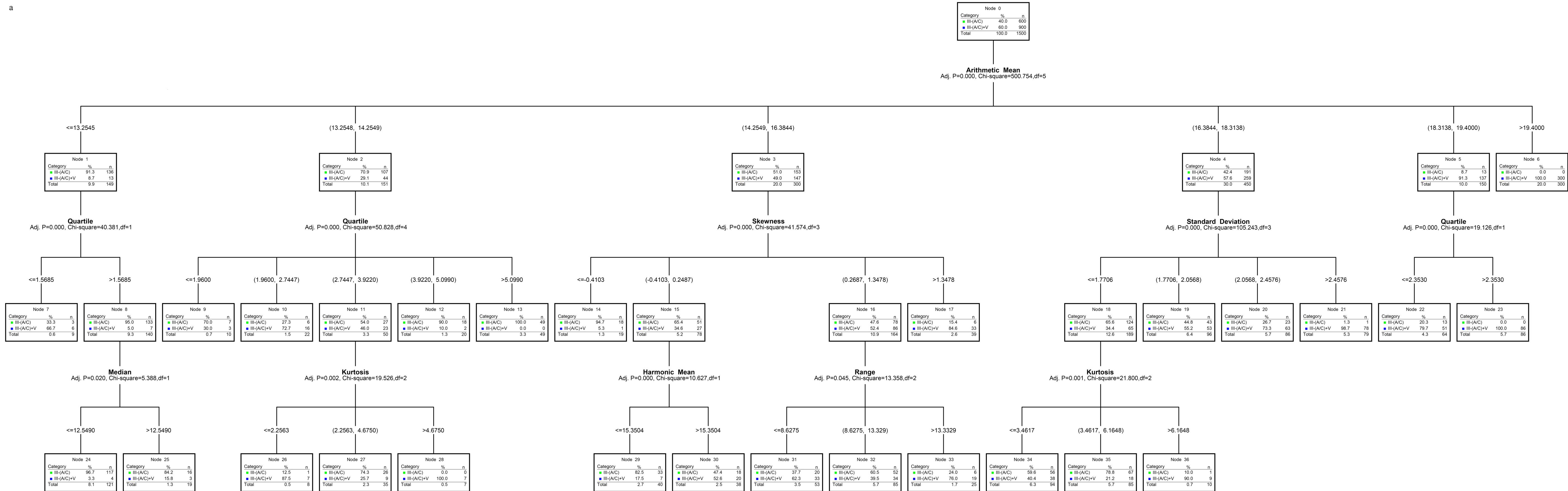

b

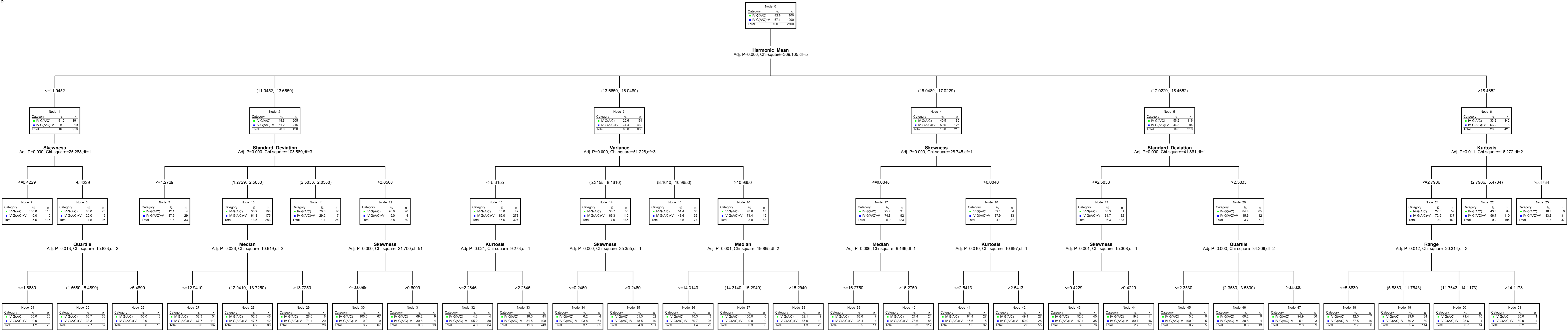

Supplement: Supplementary file 3 — Decision tree model for differentiating lupus nephritis sub-class patterns of renal pathology with CHIAD algorithm. a Class III vs class III + V. b class IV vs class IV + V. (PDF 21.5 mb) [file 12882_2017_787_MOESM3_ESM.pdf]
